# Pros and Cons of Using the Informed Basis Set to Account for Hemodynamic Response Variability with Developmental Data

**DOI:** 10.3389/fnins.2016.00322

**Published:** 2016-07-15

**Authors:** Fabien Cignetti, Emilie Salvia, Jean-Luc Anton, Marie-Hélène Grosbras, Christine Assaiante

**Affiliations:** ^1^Centre National de la Recherche Scientifique, Laboratoire de Neurosciences Cognitives UMR 7291, Aix-Marseille UniversitéMarseille, France; ^2^Centre National de la Recherche Scientifique, Fédération 3C (FR 3512), Aix-Marseille UniversitéMarseille, France; ^3^Centre National de la Recherche Scientifique, Centre IRM Fonctionnelle Cérébrale, Institut de Neurosciences de la Timone UMR 7289, Aix-Marseille UniversitéMarseille, France

**Keywords:** fMRI, development, hemodynamic response, basis set, random-effects analysis, smoothness

## Abstract

Conventional analysis of functional magnetic resonance imaging (fMRI) data using the general linear model (GLM) employs a neural model convolved with a canonical hemodynamic response function (HRF) peaking 5 s after stimulation. Incorporation of a further basis function, namely the canonical HRF temporal derivative, accounts for delays in the hemodynamic response to neural activity. A population that may benefit from this flexible approach is children whose hemodynamic response is not yet mature. Here, we examined the effects of using the set based on the canonical HRF plus its temporal derivative on both first- and second-level GLM analyses, through simulations and using developmental data (an fMRI dataset on proprioceptive mapping in children and adults). Simulations of delayed fMRI first-level data emphasized the benefit of carrying forward to the second-level a derivative boost that combines derivative and nonderivative beta estimates. In the experimental data, second-level analysis using a paired *t*-test showed increased mean amplitude estimate (i.e., increased group contrast mean) in several brain regions related to proprioceptive processing when using the derivative boost compared to using only the nonderivative term. This was true especially in children. However, carrying forward to the second-level the individual derivative boosts had adverse consequences on random-effects analysis that implemented one-sample *t*-test, yielding increased between-subject variance, thus affecting group-level statistic. Boosted data also presented a lower level of smoothness that had implication for the detection of group average activation. Imposing soft constraints on the derivative boost by limiting the time-to-peak range of the modeled response within a specified range (i.e., 4–6 s) mitigated these issues. These findings support the notion that there are pros and cons to using the informed basis set with developmental data.

## Introduction

The most common approach in fMRI today is to use a standard general linear model (GLM) regressing the blood oxygen level-dependent (BOLD) signal against predictor variables reflecting expected fluctuations due to the task, for each individual separately, and then to report group statistics (e.g., Friston et al., 1995; Worsley and Friston, 1995; Monti, 2011). To account for the indirect relationship between BOLD signal and neuronal activity, each task-related predictor variable is represented as a stick or boxcar function encoding the occurrence of an event or epoch convolved with a model of the physiological response that captures the dynamic of the vascular processes, namely a hemodynamic impulse response function (HRF). The consensus in the community is that the most suitable representation of the HRF is the sum of two gamma functions, the so-called canonical HRF, which is peaking 5 s after stimulus onset (Friston et al., 1995; Worsley and Friston, 1995). However, hemodynamic response variability (e.g., variability in response latency—Friston et al., 1998; Henson et al., 2002; Calhoun et al., 2004; Steffener et al., 2010) induces mismatches between this standard hemodynamic model and the actual data, leading to a mis-estimation of the model parameters at the individual level and introducing biases at the group-level.

There is much evidence supporting hemodynamic response variability, involving variations in BOLD signal across trials, sessions, subjects, and brain areas (Duann et al., 2002). Several works showed significant variations in the hemodynamic response across brain regions with respect to the overall shape, the time-to-onset and the time-to-peak (Henson et al., 2002; Mohamed et al., 2003; Handwerker et al., 2004; Steffener et al., 2010). Using visuo-motor tasks, Handwerker et al. (2004) and Mohamed et al. (2003) revealed time-to-onset differences between brain regions, the hemodynamic response being faster in the visual areas and peaking a few milliseconds later in motor-related areas. Handwerker et al. (2004) further reported larger between-subject variability within a given region compared to within-subject variability across regions. BOLD signal was also found to vary from one scan to another and 1 day to the next, albeit to a less extent than between-subject variability (Aguirre et al., 1998; Neumann et al., 2003). Likewise, BOLD signal magnitude variability is task-dependent, with a larger variability in active tasks compared to more passive ones (Garrett et al., 2013).

In addition, there is growing evidence that hemodynamic response variability and complexity change across the lifespan, following possibly an inverted U-shape and reaching its maximum in young adulthood (Grady, 2012). Such lower magnitude variability and complexity of BOLD signal in children and aging individuals compared to young adults might explain weaker accuracy and stability in task performance (Grady, 2012). Indeed, a less variable and complex BOLD signal likely underlies a narrower spectrum of neural states (Garrett et al., 2013). On another hand, Thomason et al. (2005) were able to reproduce children's BOLD signal by adding noise to adult data, which may indicate more marked spurious fluctuations in BOLD response to stimulations in the former compared to the latter. Within the developmental framework, a few studies also pointed out age-related changes in the shape of the hemodynamic response (Richter and Richter, 2003; Arichi et al., 2012). In particular, Arichi et al. (2012) showed a time-to-peak decrease and a peak amplitude increase from birth to adult age.

Yet, HRF variability is still not considered sufficiently in neuroimaging research. The use of basis sets instead of a single function is an appropriate approach to accommodate some variations in BOLD response across tasks, brain regions, and individuals. Using a basis set, the hemodynamic response, which is convolved with the stimulus function to model task-evoked change in BOLD signal, involves using not just a single function but a mixture of basis functions. The most popular basis set consists of the canonical HRF plus its partial derivatives with respect to delay and dispersion (Friston et al., 1998). This set, labeled informed basis set, is the most prominently used because it offers both flexibility (while precluding over-fitting) and efficiency (Friston et al., 2005a), capturing “small” variations in the latency and duration of the BOLD response using a few (two or three) basis functions. This is in contrast to other sets such as the finite impulse response (FIR) and Fourier sets that are more flexible but less powerful due to a larger set of functions to capture BOLD impulse response shape (Lindquist et al., 2009). It was also demonstrated that the informed basis set is almost identical to the principal components of variation with respect to the parameters of the Balloon model of neurovascular coupling (Friston et al., 2000), conferring biophysical validity to the set. More sophisticated basis sets/approaches have also been developed to handle variations in the onset and duration of activation but their use remains limited compared to that of the informed set (Liao et al., 2002; Friman et al., 2003; Woolrich et al., 2004; Lindquist and Wager, 2007; Lindquist et al., 2009).

The main issue regarding the informed basis set is to translate properly parameters estimates related to the canonical HRF and first/second derivative terms from individual- to group-level (Calhoun et al., 2004; Steffener et al., 2010). A popular approach is to fit the set at the individual level and to only pass up to the group-level the canonical parameter estimate. While this makes the analysis simpler, this is suboptimal as it excludes the variance related to the derivative function (i.e., the amplitude bias is not accounted for). Accordingly, Calhoun et al. (2004) proposed running group analyses using a first-level summary statistics that he called “derivative boost,” which combines derivative and nonderivative beta estimates with different weighting. Steffener et al. (2010) generalized afterwards the calculation of the derivative boost to un-normalized design matrix. Constraints on the derivative boost were also proposed to account for more or less complex BOLD response. For instance, constraining the time-to-peak of the hemodynamic response between 4 and 6 s with a set including the canonical HRF plus its temporal derivative (i.e., more weight put on the canonical term than on the derivative term in the boost) results in a BOLD response with a single peak. Loosely time-to-peak constraints (i.e., ~3–7 s) lead to either unimodal or bimodal hemodynamic response in shape.

Previous studies have already reported reduced model mis-specification and increased estimate of the BOLD response amplitude using the informed basis set—derivative boost approach instead of using the canonical HRF alone with adult data (Calhoun et al., 2004; Lindquist et al., 2009). However, we still have no indication as to whether this approach also improves amplitude estimates in populations which may show different BOLD response characteristics, such as children (Richter and Richter, 2003; Arichi et al., 2012). Besides, although there is a high likelihood of the approach to improve sensitivity at the individual-level, it is trickier to foresee its impact on group-level activation mapping. More specificity at the individual-level considering a model that combines canonical and derivatives regressors may offer a more accurate picture of activation mapping (i.e., improved fit); however, it may increase between-subject variance and impact group-level statistical testing that assesses the magnitude of an effect with respect to the variability across subjects (Penny and Holmes, 2007; Monti, 2011). Therefore, the goal of this paper is to investigate the effect of using the informed basis set to model the fMRI signal in analysis of task-based brain activation, especially in the context of special populations such as children. Our objective is not to derive complex methodological advances, but to provide elements to consider for the users of standard fMRI analysis software suites, especially SPM. As a first step, we examined the overall impact of using the informed basis set on individual simulated data. This simulation step was intended to show the benefit of translating to the second-level of analysis the derivative boost and not only the parameter estimate related to the canonical HRF. Second, we fitted the canonical HRF alone and the informed basis set (while varying the time-to-peak ranges of interest) to children and adults' data acquired during sensory (proprioceptive) stimulations. We subsequently examined the extent to which using the derivative boost impacts group-level results. Based on these results we propose guidelines for developmental neuroimaging studies.

## Materials and methods

### fMRI modeling and inference

A common approach for group fMRI data analysis consists in the two-stage summary statistics random effect model (Holmes and Friston, 1998). In the first stage, within-subject modeling assumes that changes in BOLD signal at any voxel, *y(t)*, is the output of a linear time-invariant system, expressing it as the convolution of a stimulus function, *u(t)*, and a hemodynamic response, *h(t)*:

(1) $$\begin{array}{cc} y\left(t\right)=u\left(t\right)\;⊗\;h\left(t\right) & \;(1) \end{array}$$

where, *u(t)* is a stick or boxcar function encoding the occurrence of events depending upon their durations. The hemodynamic function *h(t)* is usually assumed to be the canonical HRF. It can also be expressed as a mixture of *K* basis functions, *f*_*i*_*(t)*, to accommodate for BOLD variability:

(2) $$\begin{array}{cc} h\left(t\right)=\sum_{i=1}^{K}\beta_{i}f_{i}\left(t\right) & \;(2) \end{array}$$

where β_i_ represent the weights that determine the mixture of basis functions that best model *h(t)*. In the present study, we restricted the set to *K* = 2, including the canonical HRF plus its first temporal derivative (i.e., the informed basis set) to capture changes in the latency of the BOLD response. This choice was based on the study by Lindquist et al. (2009) who showed that including the second (dispersion) derivative provides only minor improvements in modeling. Conversion of this convolution model into a GLM is as follows:

(3a) $$\begin{array}{cc} y\left(t\right)=X\beta +\varepsilon & \;(3a) \end{array}$$

(3b) $$\begin{array}{cc} X_{i}\;=f_{i}\left(t\right)\;⊗\;u\left(t\right) & \;(3b) \end{array}$$

where *X* contains the explanatory variables (i.e., the design matrix), whose contributions to *y(t)* is determined by the parameters β that are estimated using standard least squares.

In the second stage, the individual parameter estimates (i.e., $\text{or contrast of }\hat{\beta ,\;}\text{labeled }c\hat{\beta }$) are considered as random quantities for inferences to apply at the population level. Specifically, they are combined voxel-wise into an estimator (e.g., *t*-statistic) relating the overall effect size (mean parameter estimate across subjects) to the between-subject variability, which is used to make an inference about the significance of the effect at the voxel or the cluster level (e.g., whether or not there is voxelwise or clusterwise activation in case of a one-sample *t*-test).

While the random effect model via summary statistics is in widespread use and is the default procedure in SPM, the generalized mixed-effect model is implemented by default in other software packages (e.g., FSL) to account for settings where first level variances would not be homogeneous (more generally the sphericity assumption not met), by relating the variance associated with the group effect estimate to a mixture of both within- and between-subject variability (Mumford and Nichols, 2006, 2009)^1^. However, several studies found the random-effect approach to be robust to violations of homoscedaticity (Friston et al., 2005b; Mumford and Nichols, 2009), with the approach being almost equivalent to the mixed-effect approach in the special case of second-level one sample *t*-test. Accordingly, although the present study relies on the random effect model via summary statistics implemented in the SPM package, we restricted second-level analyses to one-sample SPM models (see experimental data section below) to make outcomes the most generalizable to other fMRI packages.

### Simulations

A slow event-related design dataset including 10 s-events interspersed by 10 s-rest periods was simulated using Matlab codes adapted from Pernet (2014). Specifically, the simulated BOLD time series, *y(t)*, was obtained by convolving hypothetical neural events of various height [i.e., *u(t)*, the boxcar function] with the standard HRF [i.e., *h(t)*, the double-gamma function] using a time resolution of 0.5 s. Our simulation mimicked a slow event-related design, specifically a periodic event presented at 0.05 Hz, to approach the experimental design described later. To demonstrate the impact of adding one basis function on parameter estimates and model accuracy in case of temporal variability, events were afterwards modeled with temporal shifts (from −2 to 2 s, time step of 0.5 s) relative to the design matrix and convolved by the HRF alone vs. the HRF plus its first derivative. In the latter case, the temporal derivative was orthogonalized relative to the regressor convolved by the canonical HRF.

### Experimental data

Data are from a sensory experiment during which participants experienced vibrations of the tibialis anterior muscles (i.e., proprioceptive stimulation). The sample included 17 adults (mean age ± SD: 32.2 ± 4.5 years; 9 females) and 19 children (mean age ± SD: 8.7 ± 1.2 years; 11 females). Adult participants and parents of minors who participated in the study gave written informed consent. The study was approved by the research ethics committee CPP Sud-Méditerranée 1.

The scanning session was composed of five runs including 12-s long conditions (epochs) of vibration on the right and left tendons at 30 Hz (R30, L30) or 100 Hz (R100, L100). Each vibration condition was repeated three times per run. The order of vibration conditions was randomized within a run and REST epochs (12-s long on average) were inserted between all vibration conditions. Complete details on the protocol are provided in Cignetti et al. (2014). fMRI time series were acquired using a 3-T fMRI scanner (Medspec 30/80 AVANCE, Bruker, Ettlingen, Germany) with a T2^*^-weighted gradient echo-planar imaging sequence (42 interleaved axial slices acquisition; 3 mm thickness; 0.5 mm interslice gap; reco matrix = 64 × 64; field of view = 192 mm × 192 mm; repetition time = 2.8 s; echo time = 30 ms; flip angle = 84°). The scanning planes were parallel to the anterior commissure-posterior commissure and covered the top of the cortex down to the base of the cerebellum. Structural MRI data were also acquired using a three-dimensional T1-weighted scanning sequence (MPRAGE; repetition time = 9.4 ms; echo time = 4.4 ms; inversion time = 800 ms; field of view = 256 mm × 256 mm × 180 mm, reco matrix = 256 × 256 × 180).

Image preprocessing and statistical fMRI data analysis were conducted with SPM8 (Wellcome Department of Imaging Neuroscience, London, UK) running in Matlab 7.5 environment (Mathworks Inc., Sherbon, MA, USA) and custom-made Matlab scripts. Each run included 113 images, including 6 dummy images acquired before magnetic field saturation was reached, which were discarded. The remaining images were (i) slice-time corrected, (ii) realigned to the first image of the time series to correct for head movement between scans, (iii) unwarped to remove residual movement-related variance (Andersson et al., 2001), and (iv) co-registered to the high-resolution structural image. The structural image was normalized to the MNI T1 template image and the resulting parameters were used for spatial normalization of the functional images, which were resampled to 3-mm isotropic voxel size and smoothed with an 8-mm FWHM Gaussian kernel.

**Figure 1:**
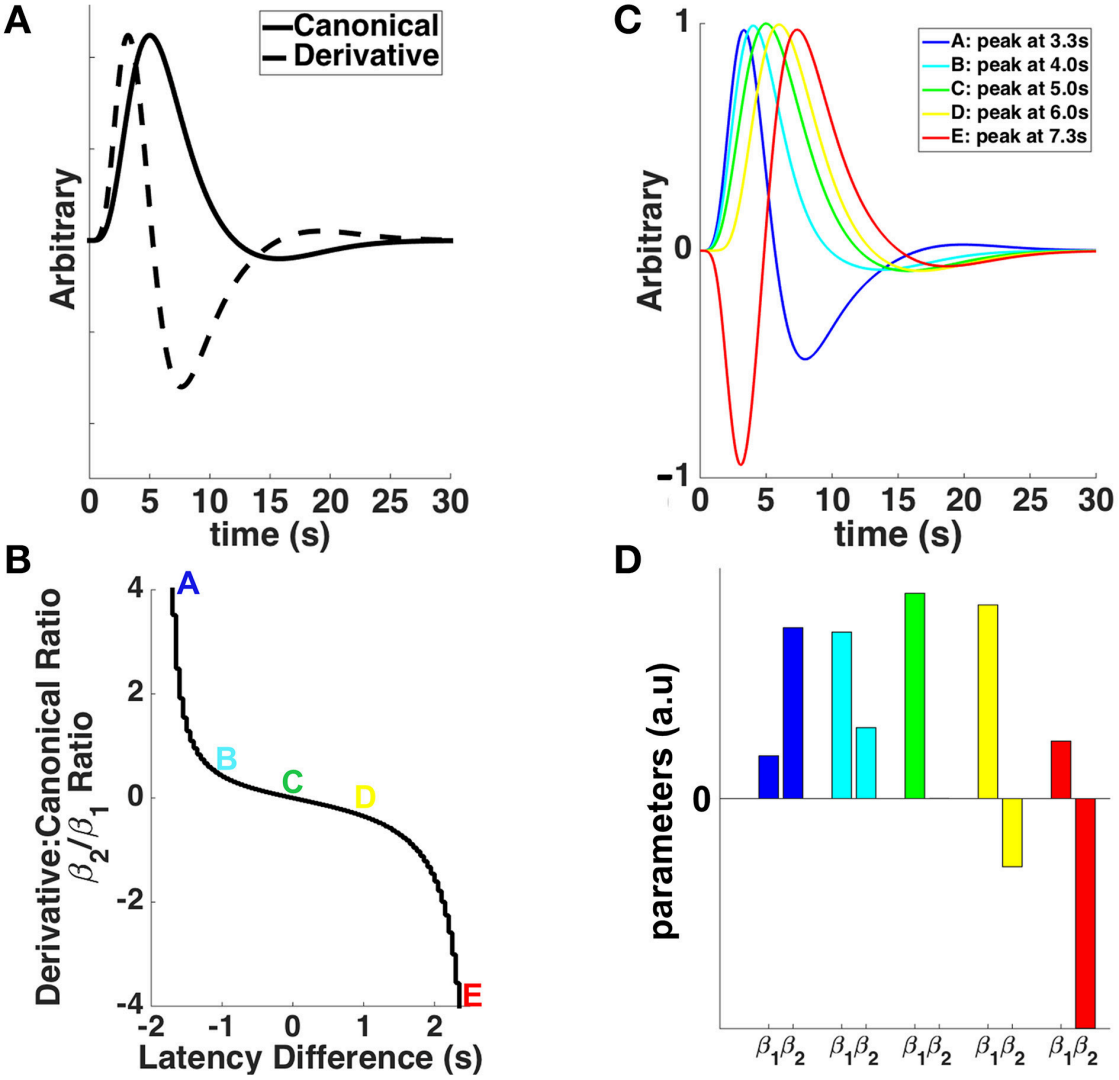
**(A)** The canonical double-gamma hemodynamic response function (HRF—solid line) and its first temporal derivative (dashed line) plotted against post-stimulus time. **(B)** The relationship between the ratios of derivative and canonical HRF parameter estimates (β_2_/β_1_) and the latency difference from the canonical HRF's time to peak (i.e., 5 s). Colored letters match with the different shapes of the HRFs shifted (or not) in time in **(C)**, and the canonical (β_1_) and derivative (β_2_) parameter estimates in **(D)**. **(C)** The canonical HRF (green), peaking at 5 s, together with HRFs shifted earlier (cyan and blue) and later (yellow and red) in time. **(D)** Parameter estimates for canonical HRF (β_1_) and temporal derivative (β_2_) associated with different shapes of the HRFs shifted (or not) in time in **(C)**.

Task-dependent changes in BOLD signal were afterwards modeled as boxcar functions time-locked to the onsets of the vibration conditions (R30, L30, R100, and L100). These regressors were convolved with either the HRF alone or the basis set and were entered into the GLM. Constant terms and realignment parameters (3 translations, 3 rotations) were also included into the GLM as covariates of no interest to account for shifting signal levels across runs and influence of head motion on BOLD signal, respectively. A high-pass filter (cutoff period = 128 s) was applied to remove low-frequency drifts in the data. Note that translational and rotational realignment estimates indicated minimal (“acceptable”) head movements in all participants including children, with values of the quality control measures broadly comparable to standards reported in previous neurodevelopmental studies (e.g., Fair et al., 2007; Kelly et al., 2009; Power et al., 2015; Cignetti et al., 2016). All participants exhibited within-run (i) maximal amplitude of translational and rotational displacements below 3 mm and 3 degrees, respectively, (ii) root mean square values for translation and rotation below 1 mm and 1 degree, respectively, and (iii) a mean framewise displacement (see Power et al., 2015 for details) below 0.2 mm. Contrast of parameter estimates were finally computed for the 100 Hz condition (i.e., sensory stimulation condition) and for the pair of conditions 100 and 30 Hz (i.e., sensory stimulation condition > sensory control condition). For the basis set, estimates related to the HRF and the temporal derivative were combined in a derivative boost (Calhoun et al., 2004; Steffener et al., 2010):

(4) $$\begin{array}{cc} H=\sqrt{{\hat{\beta_{1}}}^{2}\sum_{t=1}^{N}x_{1}{}^{2}+{\hat{\beta_{2}}}^{2}\sum_{t=1}^{N}x_{2}{}^{2}}*\frac{\hat{\beta_{1}}}{\left|\hat{\beta_{1}}\right|} & \;(4) \end{array}$$

with *H* the derivative boost, β_1_ the parameter estimate for the canonical HRF, *x*_1_ the regressor convolved with the canonical HRF, β_2_ the parameter estimate for the temporal derivative, and *x*_2_ the regressor convolved with the temporal derivative. We considered constrained and unconstrained derivative boosts, investigating a response shifted by either ±1 s relative to the canonical HRF (i.e., a time to peak between 4 and 6 s) or approximately ±2 s (i.e., the entire time interval covered by the basis set; Henson et al., 2002), respectively (Figure 1). Previous studies have shown already that the difference in the peak-time of hemodynamic responses compared to that of the canonical HRF is often greater than 1 s, extending up to 2.5 s (Handwerker et al., 2004). Therefore, it was important to consider shifts in response peak beyond ± 1 s, although we are aware that it might include more complex hemodynamic responses difficult to interpret (Calhoun et al., 2004; Steffener et al., 2010). Exact computations of the boosted contrast maps involved (i) estimating the time-to-peak of the BOLD response voxel-wise, (ii) creating a mask of the voxels whose responses peaking was within the specified temporal range (either 4–6 s or the full range of the basis set; see Figure 1), (iii) replacing the parameter estimates with their boosted counterparts for voxels within the mask, and (iv) re-estimating the contrast of interest. The core code (spmup_hrf_boost.m) we used is available at the GitHub repository: https://github.com/CPernet/spmup/blob/master/spmup_hrf_boost.m.

One-sample *t*-tests using the first-level 100 Hz and 100 minus 30 Hz contrast images were finally conducted both in children and adults, for each of the three modeling approaches (i.e., canonical HRF and basis set with temporal restriction or not). This was intended to evaluate the extent to which average group activation (reflecting here sensory mapping) was impacted by using the derivative boost. In practice, second-level one-sample *t*-test is implemented in SPM following a random-effects approach via summary statistics, which pools voxelwise the first-level contrast estimates $c{\hat{\beta }}_{1},\;\ldots ,c{\hat{\beta }}_{N}\;$from N subjects into the *t*-statistic

(5) $$\begin{array}{cc} t=c{\hat{\beta }}_{G}/\sqrt{Var\left(c{\hat{\beta }}_{G}\right)} & \;(5) \end{array}$$

where $c{\hat{\beta }}_{G}$ and $Var\left(c{\hat{\beta }}_{G}\right)$ represent the average group contrast estimate and the across-subject variance in contrast estimate, respectively (e.g., Mumford and Nichols, 2006, 2009; Penny and Holmes, 2007). ${\hat{\beta }}_{G}$ needs to be replaced by *H*_*G*_ in Equation (5) for second-level one-sample *t*-test that uses the derivative boost. After pooling the data comes the inference phase whose aim is to detect activation in the statistical images at the set-level, the cluster-level or the voxel-level (Friston et al., 1996). We considered the most popular cluster-extent inference (Hayasaka and Nichols, 2003; Woo et al., 2014), which consisted in (i) identifying clusters of contiguous voxels whose intensity exceeded a primary threshold set at the value *p* < 0.001 in our *t*-statistic images, and (ii) estimating the probability that (the spatial extent of) any of these clusters occurs as a chance process as derived using distributional approximations from the random field theory (RFT), thereby rejecting those clusters whose probability of being due to chance given the smoothness of our data was highly unlikely—or equivalently thresholding the *t*-statistic images at *p* < 0.05 family-wise error (FWE) corrected over all clusters. Although a detailed description of cluster-extent statistical inference with RFT is beyond the scope of the present study (see for details Friston et al., 1996; Hayasaka and Nichols, 2003; Nichols and Hayasaka, 2003), it is worth to mention that thresholding of statistic image with the RFT method works by calculating the smoothness of the image and then estimating the cluster-level *p*-values, a high smoothness more likely decreasing the *p*-values and revealing larger clusters of significant activation.

Hence, it results from the above steps that any change in first-level contrast estimates related to the hemodynamic response modeling strategy may affect group average activations not only through a change in the mean contrast estimate, but also through a change in the between-subject variability in contrast estimate and/or in the smoothness of the *t*-statistic image. Accordingly, we examined each of these factors separately to better interpret differences in group average activations across the modeling strategies. Differences in mean contrast estimates between the approach that used the canonical HRF and those that used the basis set (either restricted or not) were assessed using group-level paired *t*-tests on 100 Hz and 100 minus 30 Hz contrast images, whose results no longer depend on the between-subject variance (Beckmann et al., 2003). The effect of first-level modeling on between-subject variance was investigated from the denominator of Equation (5). Finally, change in data smoothness caused by first-level modeling was examined by estimating the intrinsic spatial smoothness (full width at half maximum of the theoretical Gaussian function responsible for the observed smoothness) of the individual contrast images. Our smoothness estimation (using rest_Smoothest.m; https://github.com/Chaogan-Yan/REST/blob/master/rest_Smoothest.m) relied on the algorithm described in Flitney and Jenkinson (2000).

## Results

### Simulated individual data

**Figure 2:**
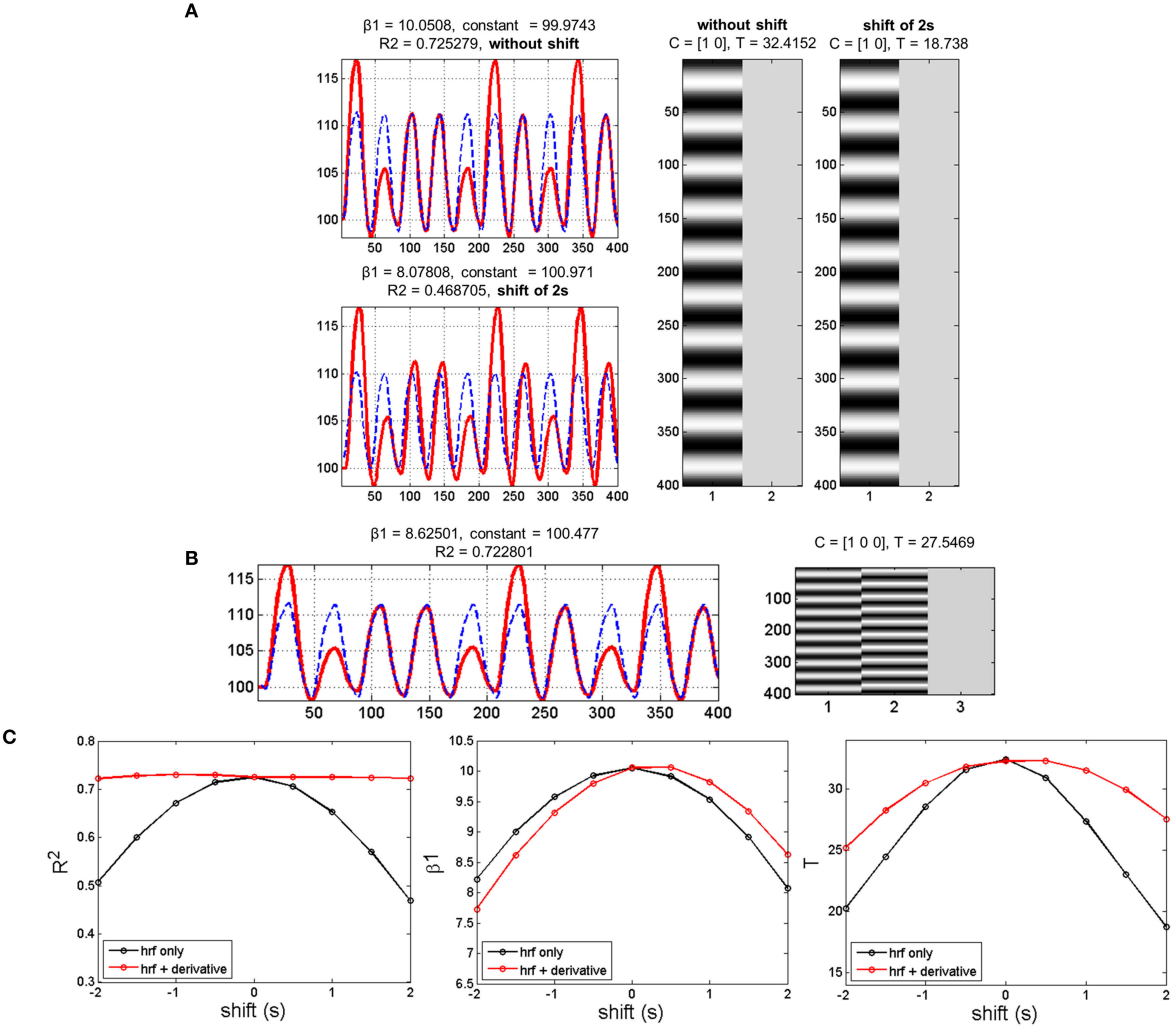
**Simulation results with data mimicking a periodic event related design. (A)** Data modeling using the HRF alone while adding or not a temporal shift of 2 s between the model (blue dashed lines) and the simulated data (red lines). Adding a temporal shift deteriorates the model fit (R^2^), the canonical HRF estimate (β1) and the T-score. **(B)** Data modeling using the HRF plus its first derivative while adding a temporal shift of 2 s between the model (blue dashed lines) and the shifted data (red lines). Adding the derivative improves substantially R^2^ and to a lower extent T-score, but has very limited impact on β1. **(C)** Variations in temporal shift from -2 to 2 s confirmed the previous observation, with the largest effect of including the first derivative being on R^2^ and the poorest effect being on β1.

The simulation showed that adding a temporal shift while modeling the data with only the canonical HRF decreased parameter estimates (canonical HRF beta estimate or β1 and T-scores) and model fitting (R^2^). The more the shift, the larger the decrease in parameter estimates and model fitting (Figures 2A,C). Using both the canonical HRF plus its first derivative to model shifted data enabled getting R^2^ at the same level as when using the canonical HRF on original (not shifted) data. Likewise, there was a beneficial effect of using the basis set on individual T-scores, although no benefit was observed with respect to β1 (see Figures 2B,C). In other words, including the derivative term together with the nonderivative term reduces deviation between the data and the model (i.e., decreased residual error), which in turn improves the T-score (i.e., the ratio between the β1 estimate and the residual error). Therefore, the rationale of decreasing the error term by regressing out the variance associated with the derivative term is valid to boost the individual T-statistics. However, it does not constitute a valid solution for second-level random-effects analysis that only relies on beta estimates, discarding first-level variance (cf. Section fMRI Modeling and Inference for details). To overcome this limit, we ran (on the experimental data) group analyses using a derivative boost that combines both nonderivative and derivative beta estimates.

### Experimental group-level data

**Figure 3:**
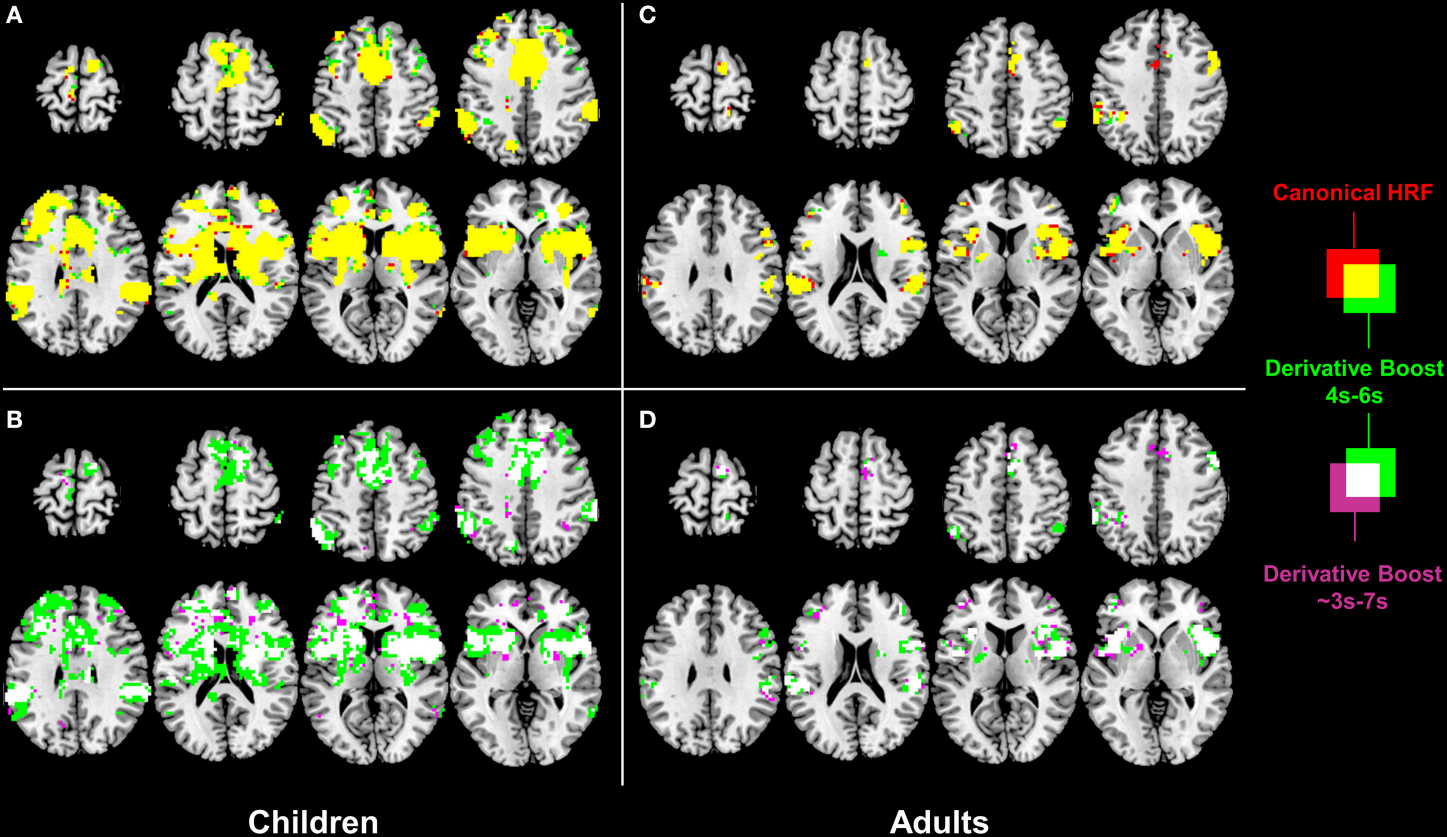
**Effect of derivative boost on proprioceptive mapping (one-sample ***t***-maps) in the 100 Hz condition. (A,C)** Mapping obtained using the canonical HRF alone (red) and the derivative boost constrained between 4 and 6 s (green) in children and adults. Overlapping active voxels (i.e., conjunction) are represented in yellow. **(B,D)** Impact of the absence of constraints on the derivative boost (i.e., the full time range covered by the basis set, as represented in purple) in children and adults. Overlapping active voxels between the two derivative boosts are represented in white. Maps are thresholded at a voxel-wise threshold of *p* < 0.001 uncorrected and a cluster extend threshold of *p* < 0.05 FWE-corrected.

One-sample *t*-tests run on the children 100 Hz contrast images revealed a similar proprioceptive mapping network topology when using eiher the canonical HRF alone or the derivative boost constrained between 4 and 6 s (Figure 3A). The network involved central (e.g., primary motor cortex), frontal (e.g., supplementary motor area, anterior cingulate cortex), parietal (e.g., inferior parietal lobule), and subcortical (e.g., putamen, thalamus) regions, which are common regions in proprioceptive processing. Extending the time interval covered by the derivative boost to 3–7 s decreased the spatial extent of the network (Figure 3B). A similar network was also found in adults when using either the canonical HRF alone or the derivative boost constrained between 4 and 6 s, although its spatial extent was restricted compared to that of the children^2^ (Figure 3C). As observed in children, the proprioceptive network of the adults became narrower when using the full derivative boost (Figure 3D). Furthermore, similar changes in proprioceptive mapping as a function of the modeling strategies were observed for the 100 minus 30 Hz contrast images (Figure S1).

**Figure 4:**
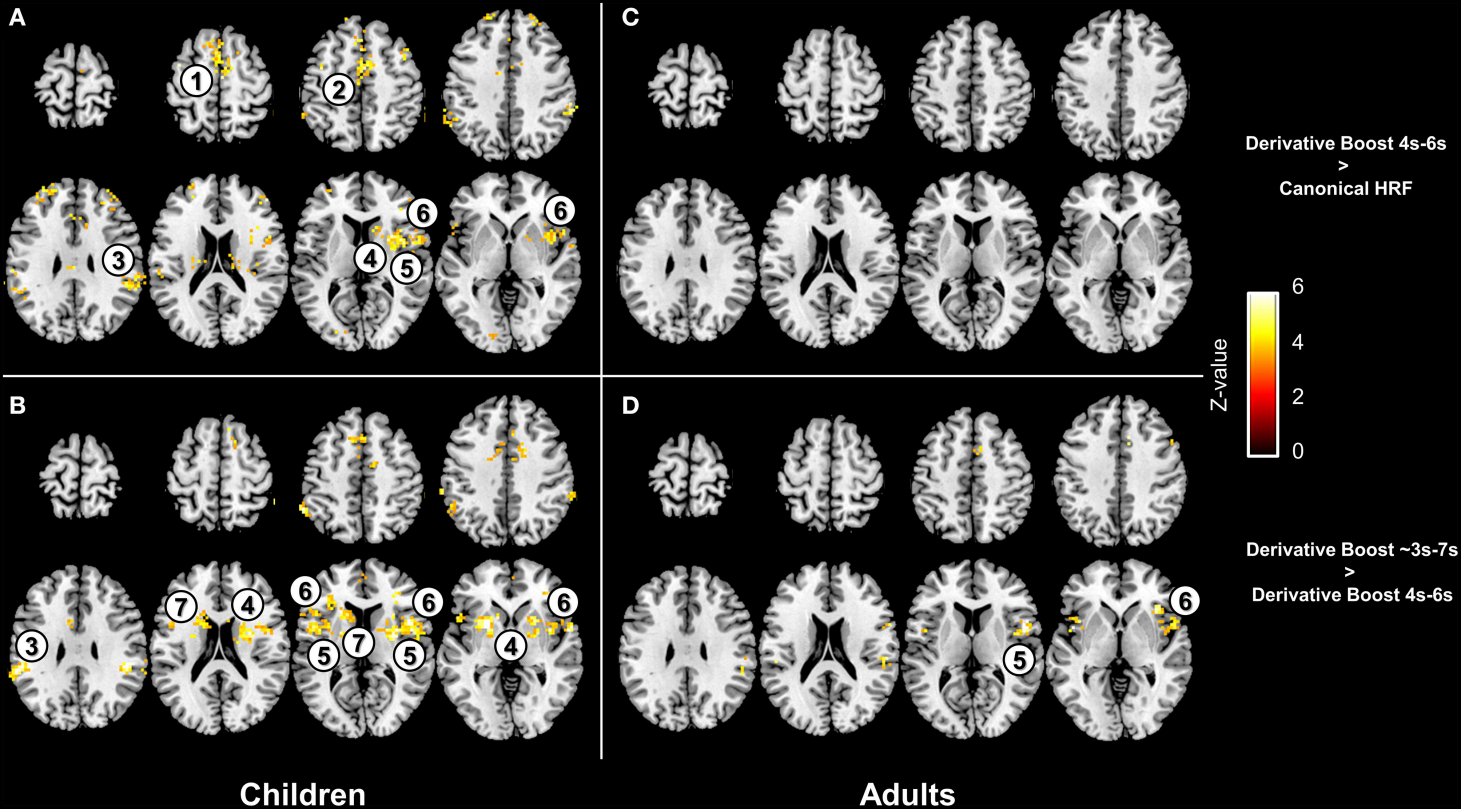
**Effect of the derivative boost on amplitude estimates in the 100 Hz proprioceptive condition**. Panel **(A,C)** display voxels where amplitude estimate is increased while using the derivative boost constrained between 4 and 6 s (as compared with the model including the canonical HRF alone) in children and adults. Panel **(B**,**D)** illustrate additional increase in amplitude estimates while using the full derivative boost compared to that constrained between 4 and 6 s, in children and adults. Maps are thresholded at a voxel-wise threshold of *p* < 0.001 uncorrected and a cluster extend threshold of *p* < 0.05 FWE-corrected. Numbers refer to main regions in which amplitude estimate was increased; 1, supplementary motor area; 2, anterior cingulate cortex; 3, inferior parietal lobule; 4, putamen; 5, anterior insula; 6, inferior frontal gyrus; 7, caudate.

Interestingly, paired *t*-tests ran on the children 100 Hz contrast images showed increased mean amplitude estimate (i.e., increased average group contrast estimate) when using the derivative boost (peak between 4 and 6 s) compared to using the canonical HRF alone. This increased amplitude was observed in several regions (e.g., anterior cingulate cortex, inferior parietal lobule, and putamen) previously identified using the one-sample *t*-tests. The full derivative boost (peak between ~3 and 7 s) led either to a further increased amplitude in some of these regions or to increased amplitude in other proprioceptive regions whose amplitude was not magnified while using the derivative boost constrained between 4 and 6 s (Figures 4A,B). In adults, there was no benefit of using the derivative boost constrained between 4 and 6 s (Figure 4C), and only limited regional gains when using the full derivative boost (Figure 4D). A similar tendency was observed for the analysis of the 100 minus 30 Hz contrast images, which is available in the supplementary data (Figure S2). Therefore, an intermediate conclusion is that using the derivative boost was beneficial with respect to average group contrast estimate—magnifying the values especially in children—while, paradoxically, there was either no improvement or adverse consequence in using it when considering group activation maps.

**Figure 5:**
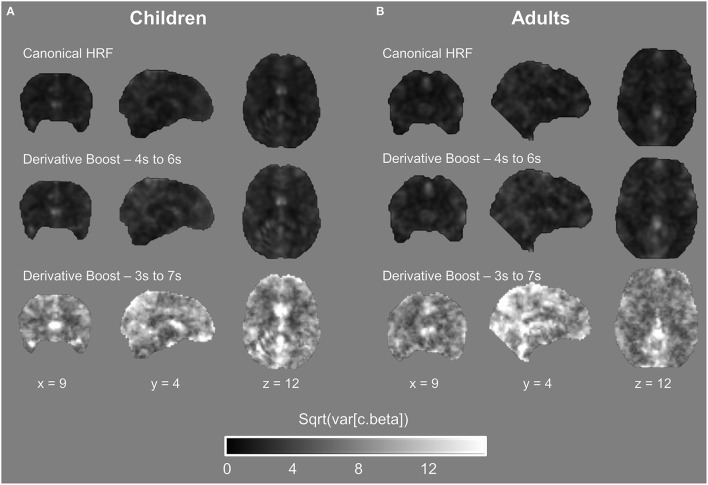
**Between-subject variability maps in the proprioceptive 100 Hz condition expressed as a function of the model used. (A,B)** Both children and adults showed an increase in variability when using the derivative boost, especially for the full derivative boost (peaking approximately between 3 and 7 s).

Importantly, the between-subject variance in contrast estimate was modulated as a function of the modeling strategy, with a slight increase of the variance for the derivative boost constrained between 4 and 6 s and a large increase of the variance for the full derivative boost (Figure 5; see also Figure S3). Recalling that test for significance using one-sample *t*-test is carried out by relating the average group contrast estimate on the between-subject variance (cf. Equation 5), the rise in the latter term therefore decreases the *t*-statistics which in turn increases cluster-level *p*-values (i.e., narrower clusters of significant activation).

**Figure 6:**
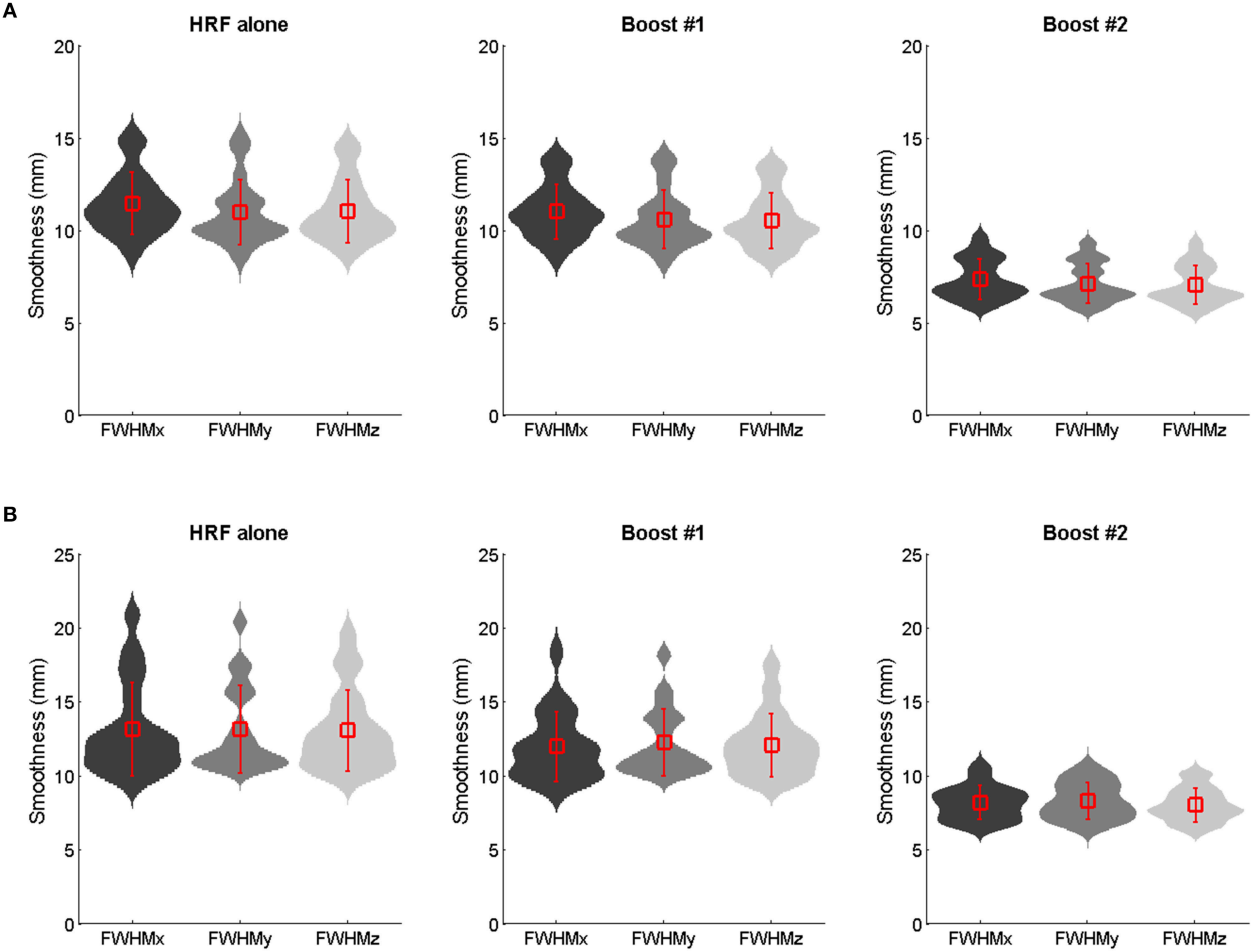
**Group mean ± SD smoothness estimates (mm full width at half maximum) of the 100 Hz contrast images as obtained using the HRF only, the constrained boost (boost #1) and the unconstrained boost (boost #2) in adults (A) and children (B)**. The violin plots show the probability density (using kernel density estimation). Reductions in smoothness occurred for boosted data, especially those boosted by means of the unconstrained basis set.

Furthermore, a lower amount of spatial smoothness was found in the boosted contrast images, especially in those generated using the unconstrained boost (Figure 6; see also Figure S4), which also contributed to increase the likelihood of finding clusters of significant activation smaller in size.

## Discussion

The present study falls within the framework of examining the extent to which implementation of the informed basis set is relevant for modeling task-evoked BOLD responses (e.g., Calhoun et al., 2004; Lindquist et al., 2009; Steffener et al., 2010), when considering developmental data (children vs. adults data). Simulations of fMRI individual data demonstrated that incorporating the canonical HRF plus its temporal derivative into the hemodynamic model is meaningless in case only beta estimate related to the canonical HRF would be used at group level, as expected (Calhoun et al., 2004). More importantly, findings showed pros and cons of using the derivative boost at the second level. Therefore, accounting for hemodynamic response variability at the group level using the informed basis set has complex consequences that need to be discussed in details.

### Pros of using the derivative boost

Several cortical (e.g., anterior cingulate cortex, supplementary motor area, inferior frontal gyrus, inferior parietal lobule) and subcortical (e.g., putamen) regions typically involved in proprioceptive processing (e.g., Goble et al., 2011, 2012; Cignetti et al., 2014) showed increased amplitude values of parameter estimates when combining derivative and nonderivative terms in the derivative boost, particularly in children (see Figure 4 and Figure S2). This finding confirms the potential of using the derivative boost to account for delays in the hemodynamic responses in adults, eventually reaching their peak amplitudes earlier or later than the canonical HRF's peak of 5 s (Henson et al., 2002; Calhoun et al., 2004; Lindquist et al., 2009; Steffener et al., 2010). It also indicates more systematic delays in the time-to-peak amplitude in children compared to adults, which seems consistent with previous developmental studies having reported age-related changes in the shape of the BOLD response, and especially in the time taken to reach the positive peak of the HRF that would be longer earlier in life (Richter and Richter, 2003; Arichi et al., 2012). Besides, there is evidence of noisier BOLD responses in children than in adults, or in other words more fluctuations in the BOLD signal changes in response to stimulations (Thomason et al., 2005). Accordingly, the stronger benefits in children of having used the derivative boost may not only relate to a hemodynamic response time course that is different from that of the canonical HRF (i.e., a response shifted in time) but also to a BOLD signal that is more variable in time (i.e., a response less stable and less well-calibrated to the inputs) at younger ages.

Although speculative, we can mention several factors that likely subtend changes in the BOLD-evoked response during development, and ultimately calls for the use of flexible basis sets, here as the informed basis set, to model neurodevelopmental fMRI data. A main factor would be age-related change in cerebral blood flow, whose localized increase is known to be the key to the positive peak of the BOLD response (Buxton et al., 2004; Chen and Pike, 2009a,b). However, the physiology of the BOLD response is complex and current findings support a picture where not only cerebral blood flow but also cerebral metabolic rate of oxygen, both driven by different aspects of neural activity (e.g., synaptic activity, spiking, neurotransmitters, neuromodulators), contribute to the BOLD signal (Buxton, 2012). Therefore, it is likely that all of these factors mature with age and experience and as such play a key role in the larger deviations from the canonical HRF in children. However, although using the derivative boost effectively increased individual amplitude estimates and magnified the mean effect over subjects, any conclusion requires also considering between-subject variability (i.e., second-level random effects) and data smoothness.

### Cons of using the derivative boost

An important finding was that using the derivative boost did not magnify the proprioceptive network for a latency shift relative to the canonical HRF of ±1 s (Figures 3A–C and Figures S1A–C), and even deteriorated it for a latency of ±2 s (Figures 3B–D and Figures S1B–D) in both children and adults. Such a result was found to be related to an increased between-subject variability of the amplitude estimate, the increment in the between-subject variability being the most important for the model allowing the hemodynamic response to be shifted up to approximately ±2 s (Figure 5 and Figure S3). This indicates that the more flexible the model was in term of the fitted response, the more the amplitude estimates varied between the subjects and affected the *t*-statistics. This is a direct consequence of the random-effect approach where statistical testing is equivalent to examining whether the magnitude of an effect is significant with respect to the variability across subjects (e.g., Friston et al., 2002; Mumford and Nichols, 2006, 2009; Mumford and Poldrack, 2007; Penny and Holmes, 2007; Monti, 2011). Thus, although using the informed basis set is a common strategy to increase model flexibility or sensitivity while adequately controlling for power consumption and the risk of over-fitting (e.g., Lindquist et al., 2009; Monti, 2011), there is also a cost in using it when dealing with multiple subjects' analysis, namely an increase in the likelihood of a rise in subject-to-subject variation. The use of the basis set also affected the amount of smoothness (FWHM) of the fMRI data conveyed to the second-level of analysis, with a significant decrease of smoothness especially when no constraints were applied on the basis set. Given that smoothness is a key parameter for the RFT based *p*-values—a reduction in smoothness increasing the corrected *p*-values and decreasing significance (Nichols and Hayasaka, 2003)—this outcome is problematic. Besides, the recommended rule of thumb is three voxels FWHM smoothness (here, FWHM 9 mm) for the RFT theory to work correctly (Petersson et al., 1999; Nichols and Hayasaka, 2003), which was only marginally met when using the full derivative boost (Figure 6).

Therefore, the best case scenario in using the informed basis set-derivative boost approach requires imposing soft constraints (i.e., response peak between 4 and 6 s) to the full model (i.e., nonderivative and derivative together) fitting the actual data. Under such condition, one may expect the approach to outperform the most common canonical HRF approach at the individual level and to achieve good network detection at the group-level. Limiting hemodynamic responses peaking within the 4–6 s range also avoid dealing with physiologically ambiguous results, such as responses with a bimodal shape (Calhoun et al., 2004; Steffener et al., 2010).

### Limitations and future directions

A limit of the study relates to design considerations. Focus was only on a slow event-related design and thus the generalizability of the outcomes to other designs, including block and fast event-related designs, remains to be explored. If we consider the mean contrast estimate over subjects, it is reasonable to expect a beneficial effect (i.e., increased estimate) of the informed basis set-derivative boost approach in any design, the effect being likely the least important with block design data and the most important with fast event-related design data (Aguirre et al., 1998; Hopfinger et al., 2000). However, making predictions on its potential impact on second-level random effects corresponding to between-subject variability is a far more delicate matter that requires further studies.

Our modeling strategy was also restricted to the informed basis set while many other models, more or less flexible, could have been tested (e.g., Lindquist et al., 2009). Although our results argue against using too much flexible models (such as the FIR model) when examining group activation, these models that better fit individual data could nevertheless be used to better characterize the shape of the hemodynamic response in children. Likewise, flexible basis sets are likely to be relevant for brain/behavior correlational studies, where focus is put not on mean (group) effect size but on variations of the effect across subjects. On the other hand, approaches that use group-level HRF profile to model individual data would take better account of between-subject variability and may lead to less biased group-level statistics (Vincent et al., 2014).

Another note has to do with the assumptions made by the random effects model used in the present study (implemented by default in SPM), which assumes that first-level variances are homogeneous and that second-level effect estimates follow a Gaussian distribution (i.e., no outliers). However, improving model fitting at the fist-level using the informed basis set may decrease the likelihood of these assumptions being correct. An alternative would be to use mixed-effects models that should work reasonably well if the previous assumptions do not hold, down-weighting outliers and subjects with relatively high intrasubject variability at the second level. Thus, a mixed effects model may be theoretically capable of better controlling the rise in across-subject variations in effect magnitude induced by the informed basis set-derivative boost approach. In practice, however, studies showed very modest improvement in group-level statistics using the mixed-effects model compared to the random-effects model (Beckmann et al., 2003; Friston et al., 2005b; Mumford and Nichols, 2009; Chen et al., 2012). Using outlier-induced heteroscedastic data, Mumford and Nichols (2009) even demonstrated that the two models are almost equivalent for second-level one-sample *t*-tests as used in the present study. Accordingly, we feel confident that incorporating the informed basis set to the mixed effects model should affect group-level statistics in a close way as for the random effects model. This said, investigating empirically this issue is left for future research.

Finally, we used the most popular RFT method for (cluster-extent) thresholding our statistical maps. This method requires the images to be sufficiently smooth (~FWHM 3 voxels), which was met when using the recommended constrained basis set (despite a slight decrease in smoothness). However, depending on the Gaussian kernel smoothing applied as part of preprocessing, this criteria might not be met. Applying kernel smoothing on boosted data and not on preprocessed data may constitute a more adequate solution to meet smoothness RFT prerequisite. This said, one should always check the estimated FWHM of their analysis and consider permutation methods in case the images are not sufficiently smooth (Hayasaka and Nichols, 2003).

## Conclusion

The present study demonstrates that using the informed basis set-derivative boost approach captured individual variance in the hemodynamic responses better, especially in children whose mean amplitude estimate (i.e., the group mean estimate) were significantly increased over several brain regions. However, such an approach had adverse consequences on random effects analysis for a group contrast mean (i.e., second level one-sample *t*-test), where the group-level statistics suffered from the increased subject-to-subject variability and decreased data smoothness. Limiting the time-to-peak range of the modeled response between 4 and 6 s (i.e., imposing soft constraints on the basis set) appeared as an effective solution that mitigated these drawbacks. Exploring solutions to make the approach more suitable for random effects analysis at the second level is needed before drawing any firm conclusion on its usefulness with developmental fMRI data.

## Author contributions

Conceived and designed the experiment: FC, CA. Acquisition of data: FC, CA. Analysis and interpretation of data: FC, ES, JA, MG, CA. Drafting the work: FC, ES, JA, MG, CA. Final approval of the work: FC, ES, JA, MG, CA. Being accountable for the accuracy and integrity of the work: FC, ES, JA, MG, CA.

### Conflict of interest statement

The authors declare that the research was conducted in the absence of any commercial or financial relationships that could be construed as a potential conflict of interest.

## Footnotes

^1^Note, however, that mixed effects model is also available in SPM through the spm_mfx.m function.

^2^The spatial contraction of the activation patterns from childhood to adulthood is a typical phenomenon of functional brain networks development, likely related to synaptic pruning and experience-dependent modulation of functional wiring (e.g., Durston et al., 2006; Kelly et al., 2009; Cignetti et al., 2016). Thus, this result does not mark a lack of sensitivity of the analysis that would have occurred with adult data.
