# Supplementary material for: Pros and Cons of Using the Informed Basis Set to Account for Hemodynamic Response Variability with Developmental Data
Source: Front Neurosci. 2016 Jul 15;10:322. doi: 10.3389/fnins.2016.00322 (PMC4945642; doi:10.3389/fnins.2016.00322)
Supplement: Supplementary file 1 [file DataSheet1.DOCX]

**Supplementary figure legends**

Figure S1

**Effect of derivative boost on proprioceptive mapping (one-sample t-maps) in the 100 Hz minus 30 Hz condition**. **A.** and **C.**: Mapping obtained using the canonical HRF alone (red) and the derivative boost constrained between 4s and 6s (green) in children and adults. Overlapping active voxels (i.e. conjunction) are represented in yellow. **B.** and **D.** Impact of the absence of constraints on the derivative boost (i.e. the full time range covered by the basis set, as represented in purple) in children and adults. Overlapping active voxels between the two derivative boosts are represented in white. Maps are thresholded at a voxel-wise threshold of p < 0.001 uncorrected and a cluster extend threshold of p < 0.05 FWE-corrected.

Figure S2

**Effect of the derivative boost on amplitude estimates in the 100 Hz minus 30 Hz proprioceptive condition.** **A.** and **C.** display voxels where amplitude estimate is increased while using the derivative boost constrained between 4s and 6s (as compared with the model including the canonical HRF alone) in children and adults. **B.** and **D.** illustrate additional increase in amplitude estimates while using the full derivative boost compared to that constrained between 4s and 6s, in children and adults. Maps are thresholded at a voxel-wise threshold of p < 0.001 uncorrected and a cluster extend threshold of p < 0.05 FWE-corrected. Numbers refer to main regions in which amplitude estimate was increased; 1: anterior cingulate cortex; 2: anterior insula; 3: inferior frontal gyrus.

Figure S3

**Between-subject variability maps in the proprioceptive 100 Hz minus 30 Hz condition expressed as a function of the model used. A.** and **B.**: Both children and adults showed an increase in variability when using the derivative boost, especially for the full derivative boost (peaking approximately between 3s and 7s).

Figure S4

**Group mean ± SD smoothness estimates (mm full width at half maximum) of the 100 Hz minus 30 Hz contrast images as obtained using the HRF only, the constrained boost (boost #1) and the unconstrained boost (boost #2). The violin plots show the probability density (using kernel density estimation).** Reductions in smoothness occurred for boosted data, especially those boosted by means of the unconstrained basis set.
