# Supplementary figures and images for: Pros and Cons of Using the Informed Basis Set to Account for Hemodynamic Response Variability with Developmental Data
Source: Front Neurosci. 2016 Jul 15;10:322. doi: 10.3389/fnins.2016.00322 (PMC4945642; doi:10.3389/fnins.2016.00322)

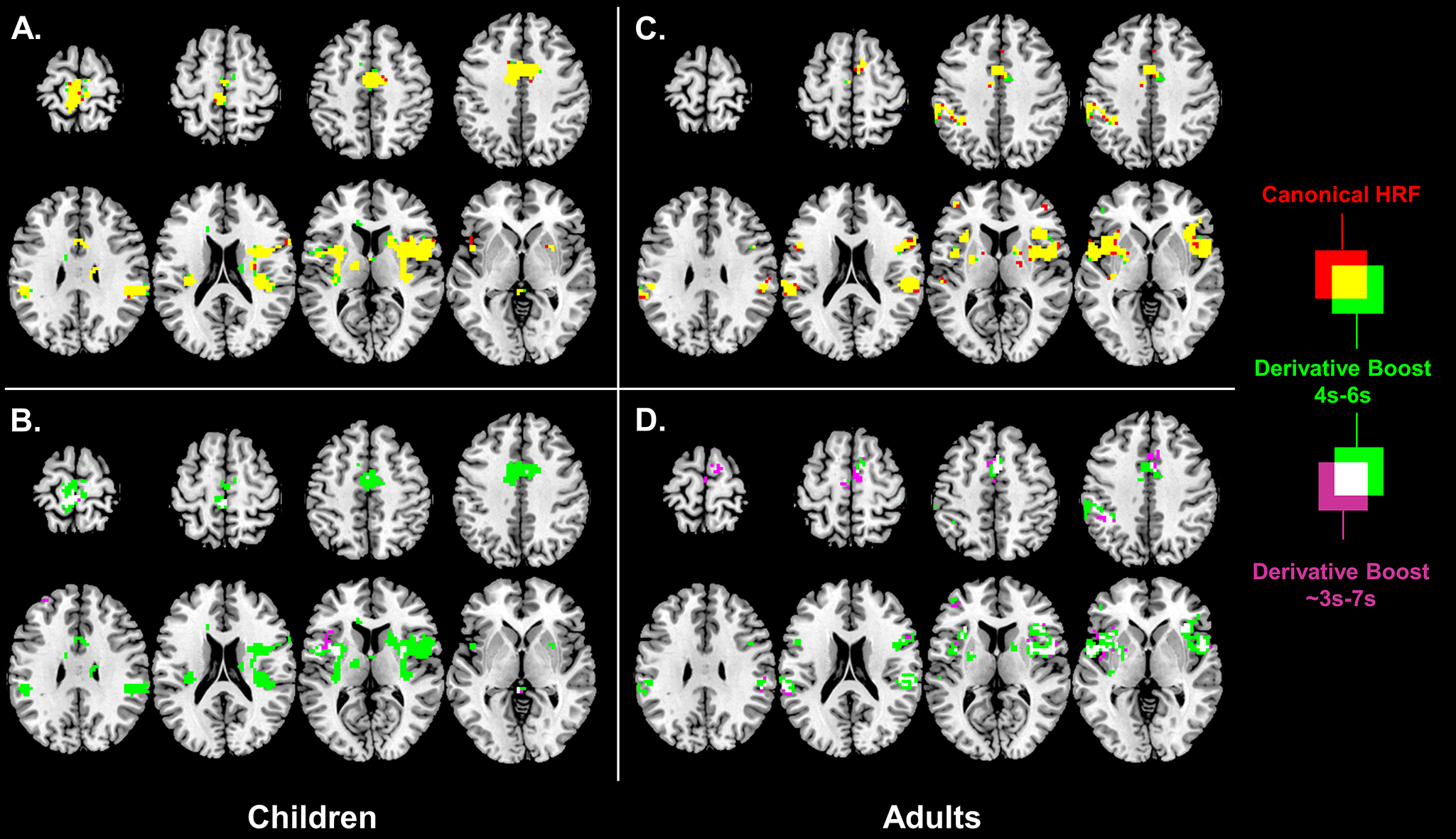

Supplement: Supplementary file 2 [file Image1.TIF]

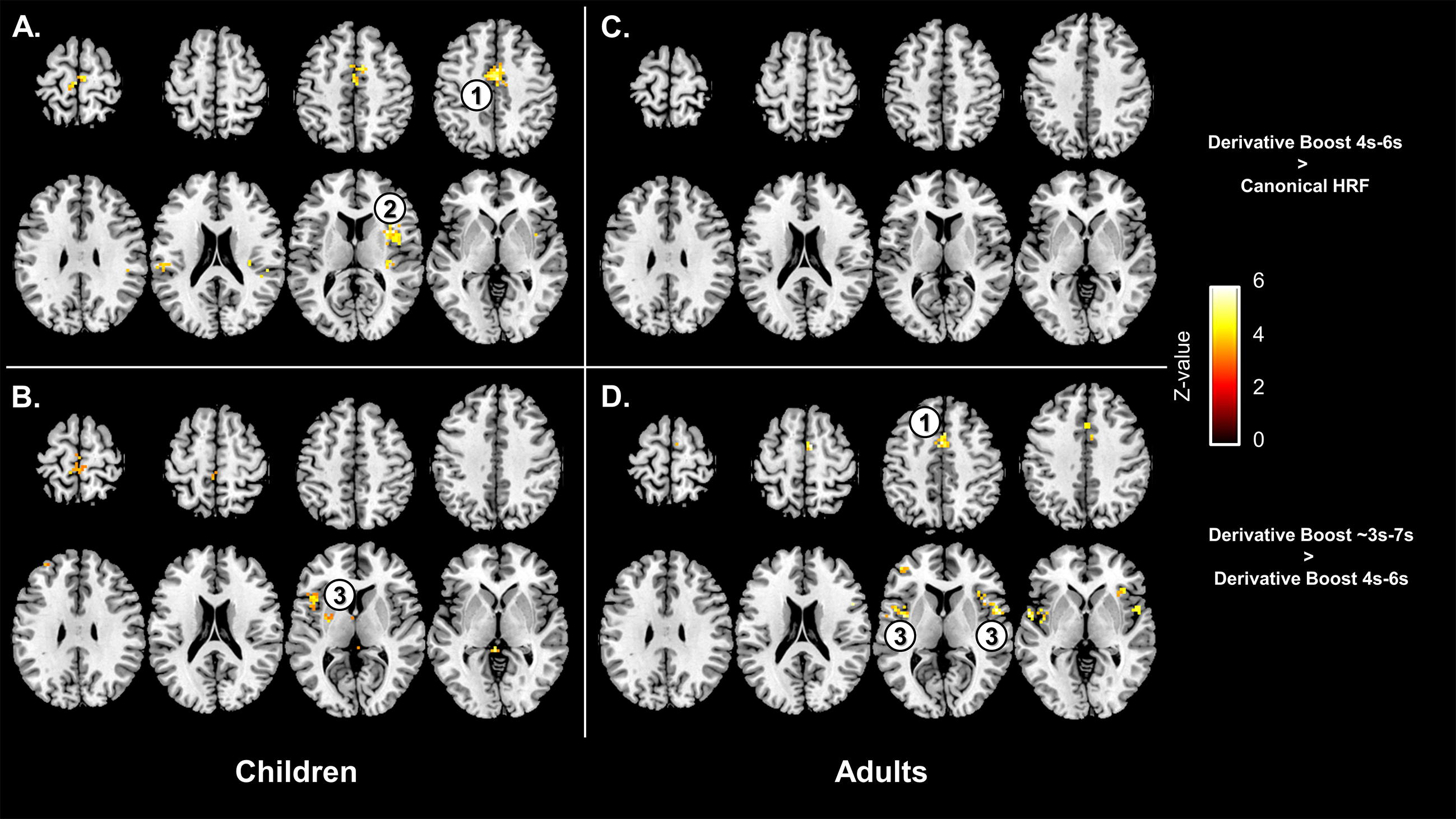

Supplement: Supplementary file 3 [file Image2.TIF]

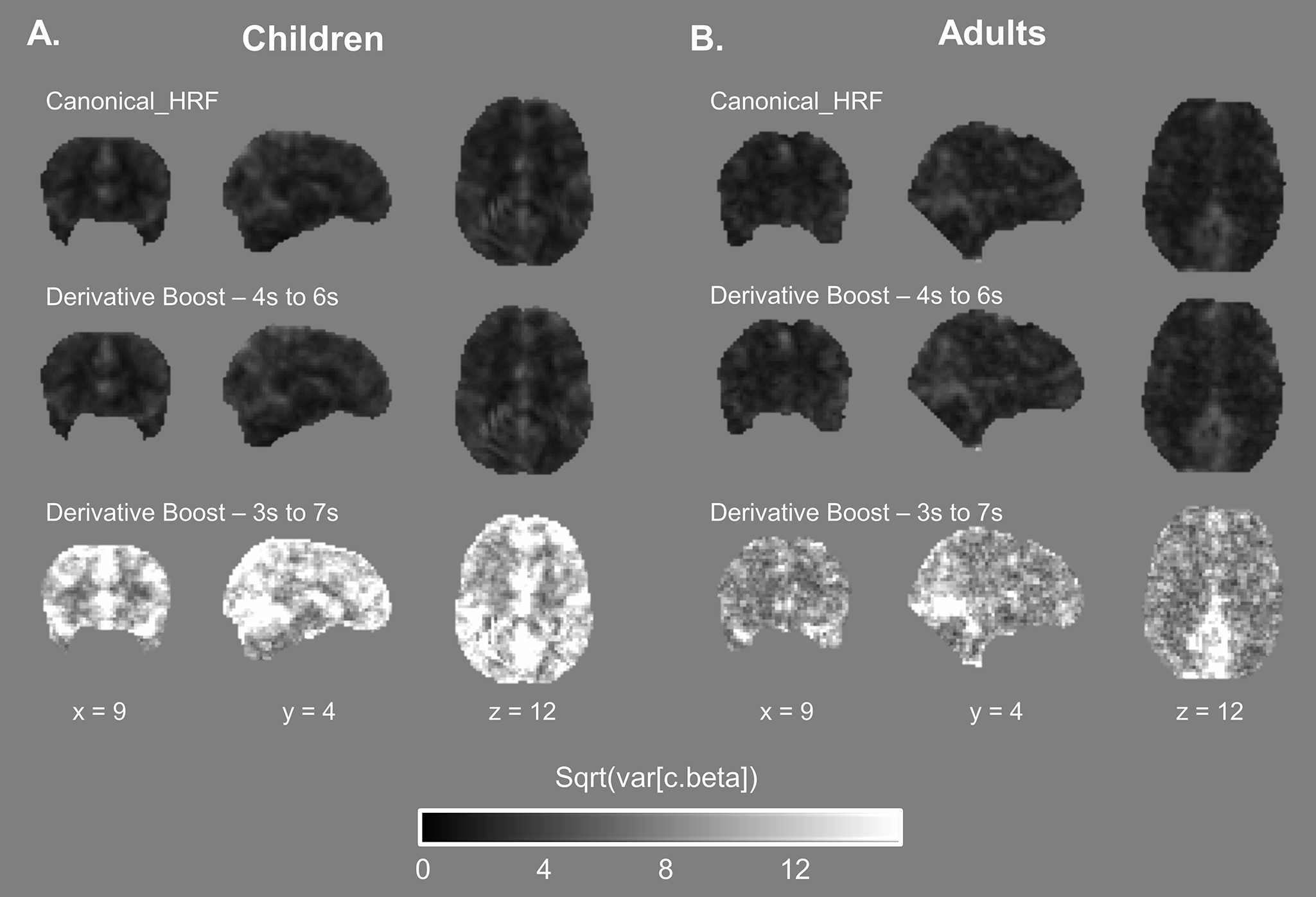

Supplement: Supplementary file 4 [file Image3.TIF]

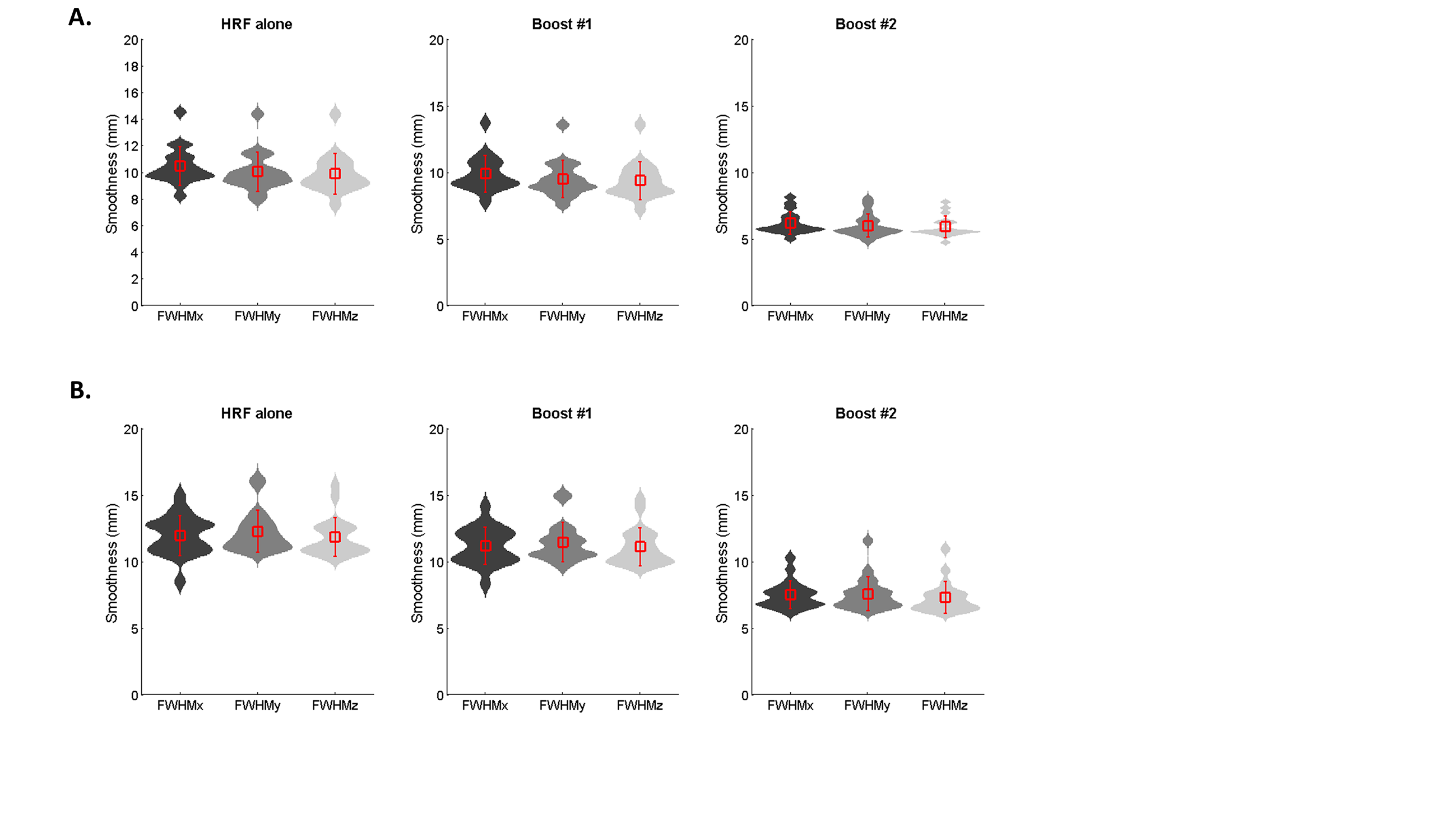

Supplement: Supplementary file 5 [file Image4.TIF]
